# Aggresome formation is regulated by RanBPM through an interaction with HDAC6

**DOI:** 10.1242/bio.20147021

**Published:** 2014-05-02

**Authors:** Louisa M. Salemi, Ahmad W. Almawi, Karen J. Lefebvre, Caroline Schild-Poulter

**Affiliations:** 1 Robarts Research Institute, The University of Western Ontario, London, ON N6A 5B7, Canada; 2 Department of Biochemistry, Schulich School of Medicine and Dentistry, The University of Western Ontario, London, ON N6A 5C1, Canada

**Keywords:** Aggresome, RanBPM, HDAC6, Proteasome inhibition, DNA damage

## Abstract

In conditions of proteasomal impairment, the build-up of damaged or misfolded proteins activates a cellular response leading to the recruitment of damaged proteins into perinuclear aggregates called aggresomes. Aggresome formation involves the retrograde transport of cargo proteins along the microtubule network and is dependent on the histone deacetylase HDAC6. Here we show that ionizing radiation (IR) promotes Ran-Binding Protein M (RanBPM) relocalization into discrete perinuclear foci where it co-localizes with aggresome components ubiquitin, dynein and HDAC6, suggesting that the RanBPM perinuclear clusters correspond to aggresomes. RanBPM was also recruited to aggresomes following treatment with the proteasome inhibitor MG132 and the DNA-damaging agent etoposide. Strikingly, aggresome formation by HDAC6 was markedly impaired in RanBPM shRNA cells, but was restored by re-expression of RanBPM. RanBPM was found to interact with HDAC6 and to inhibit its deacetylase activity. This interaction was abrogated by a RanBPM deletion of its LisH/CTLH domain, which also prevented aggresome formation, suggesting that RanBPM promotes aggresome formation through an association with HDAC6. Our results suggest that RanBPM regulates HDAC6 activity and is a central regulator of aggresome formation.

## INTRODUCTION

Misfolded proteins are generally processed by chaperone-mediated refolding or by proteasomal degradation through the ubiquitin–proteasome system (UPS) (Schwartz and Ciechanover, 2009; Wójcik and DeMartino, 2003). In conditions where these systems are impaired or overwhelmed, misfolded proteins accumulate in perinuclear structures called aggresomes (Garcia-Mata et al., 2002; Kopito, 2000; Wójcik and DeMartino, 2003). Unfolded/misfolded proteins are transported from throughout the cell to the aggresome via a dynein-dependent retrograde transport along the microtubule network. The formation of aggresomes can be induced by proteasome inhibitors (such as MG132) and also by overexpression of various proteins (García-Mata et al., 1999; Garcia-Mata et al., 2002; Lehotzky et al., 2004). In addition to aggregated proteins, aggresomes recruit several other components, including chaperones, for instance heat shock protein 70 (Hsp70), ubiquitin and ubiquitination enzymes such as ataxin 3 (AT3) and carboxy terminus of Hsp70-interacting protein (CHIP), as well as proteasome components and motor proteins such as dynein and dynamitin (Garcia-Mata et al., 2002; Johnston et al., 2002; Chin et al., 2008; Rodriguez-Gonzalez et al., 2008; Yao, 2010; Zhang and Qian, 2011). Recently, the histone deacetylase HDAC6 has been shown to be an essential component of the aggresome pathway, by functioning as a key factor recruiting protein cargo to the dynein motor for transport into the aggresome and by regulating a cell response pathway involving the activation of a heat-shock response that helps the clearance of the aggregates (Boyault et al., 2007b; Kawaguchi et al., 2003). Other components that appear to be essential to aggresome formation include the chaperones CHIP and Hsp70, as well as protein kinase CK2 which has recently been shown to regulate HDAC6 activity through phosphorylation (Sha et al., 2009; Watabe and Nakaki, 2011; Zhang and Qian, 2011).

The relationship between protein aggregation and cell death is still a matter of debate, as both protective and death-inducing functions have been suggested for aggresome-like structures (Garcia-Mata et al., 2002; Kopito, 2000). Aggresome formation is generally recognized as a protective response from the cell to an otherwise toxic build-up of abnormal/unfolded proteins. However, it has also been concluded that aggresomes can be toxic and induce apoptosis if the aggregated substrates cannot be processed (Bennett et al., 2005; Garcia-Mata et al., 2002; Kristiansen et al., 2005; Rantanen et al., 2008; Tanaka et al., 2004; Wójcik and DeMartino, 2003; Wójcik et al., 2004). Finally, while aggresomes have raised considerable interest as a hallmark of neurodegenerative diseases, they have also more recently attracted attention in the cancer field because of the link between aggresomes and the UPS (Dahlmann, 2007; Rodriguez-Gonzalez et al., 2008). Proteasome inhibitors (such as Bortezomib) have recently emerged as promising therapeutic agents in the treatment of some cancers (Orlowski and Kuhn, 2008). However, our understanding of aggresome formation and regulation as well as their role in regulating cell viability, which is crucial to understand how these drugs function, remains limited.

RanBPM (Ran-binding protein M, also called RanBP9) is a ubiquitous, nucleocytoplasmic and evolutionary conserved protein whose function is largely unknown. RanBPM contains several conserved domains including a SPla/Ryanodine receptor (SPRY), a protein interaction module (Perfetto et al., 2013), a lissencephaly type-1-like homology (LisH) motif suggested to function as a dimerization domain and a microtubule-binding domain (Emes and Ponting, 2001; Kim et al., 2004), and a carboxy-terminal to LisH (CTLH) domain of unknown function (Emes and Ponting, 2001). RanBPM was originally identified in a yeast two-hybrid screen as a protein interacting with the transport protein Ran (Nakamura et al., 1998). However, the interaction was not confirmed and its involvement in nucleocytoplasmic transport was not substantiated (Nishitani et al., 2001). Subsequently, RanBPM was reported to interact with various proteins, including cytoplasmic kinases, steroid receptors and transcription factors, and was suggested to participate in various cellular processes such as cell growth and cell migration signaling (Hafizi et al., 2005; Valiyaveettil et al., 2008; Zou et al., 2003), neuronal morphogenesis (Brunkhorst et al., 2005; Chang et al., 2010; Togashi et al., 2006) and the regulation of gene transcription (Brunkhorst et al., 2005; Poirier et al., 2006). Several studies have also suggested RanBPM to be present in a large multiprotein complex and to function as an adaptor or a scaffolding protein (Kobayashi et al., 2007; Nishitani et al., 2001; Umeda et al., 2003). Additionally, a function for RanBPM in regulating apoptosis has been suggested based on its interaction with factors implicated in apoptotic pathways (Bai et al., 2003; Kramer et al., 2005; Mikolajczyk et al., 2003; Wang et al., 2002). The generation of RanBPM-deficient mice has recently revealed a role for RanBPM in male and female gametogenesis; however, additional defects resulting from RanBPM deficiency remain to be investigated (Puverel et al., 2011).

Our previous investigations have provided evidence that RanBPM functions as an activator of apoptotic pathways and regulates the activation of apoptosis induced by DNA damage (Atabakhsh et al., 2009). We showed that siRNA-directed knockdown of RanBPM prevented DNA damage-induced apoptosis and promoted cell survival in response to ionizing radiation (IR). RanBPM shRNA cells displayed a sharp decrease of mitochondria-associated Bax protein levels, whereas Bcl-2 levels were dramatically up-regulated, providing a novel function for RanBPM in the regulation of DNA damage-induced apoptosis through regulation of the mitochondrial apoptotic pathway. In addition, following IR treatment, we observed the relocalization of RanBPM from the nucleus to the cytoplasm, suggesting that the activation of apoptotic pathways by RanBPM in response to DNA damage may be regulated by nucleocytoplasmic trafficking (Atabakhsh et al., 2009).

In follow-up studies aimed at characterizing IR-induced RanBPM relocalization, we found that RanBPM clustered into discrete perinuclear foci where it co-localized with ubiquitin, dynein and HDAC6, revealing that these RanBPM aggregates correspond to aggresomes. We show that RanBPM is also recruited to aggresomes in response to the proteasome inhibitor MG132 and the DNA-damaging agent etoposide. In addition we show that RanBPM is essential for aggresome formation and that this function is dependent on the RanBPM LisH/CTLH domain which mediates its interaction with HDAC6. Our work suggests that RanBPM regulates HDAC6 activity and is a central regulator of aggresome formation.

## RESULTS

### RanBPM is recruited to aggresomes in response to IR and proteasome inhibition

**Fig. 1.**
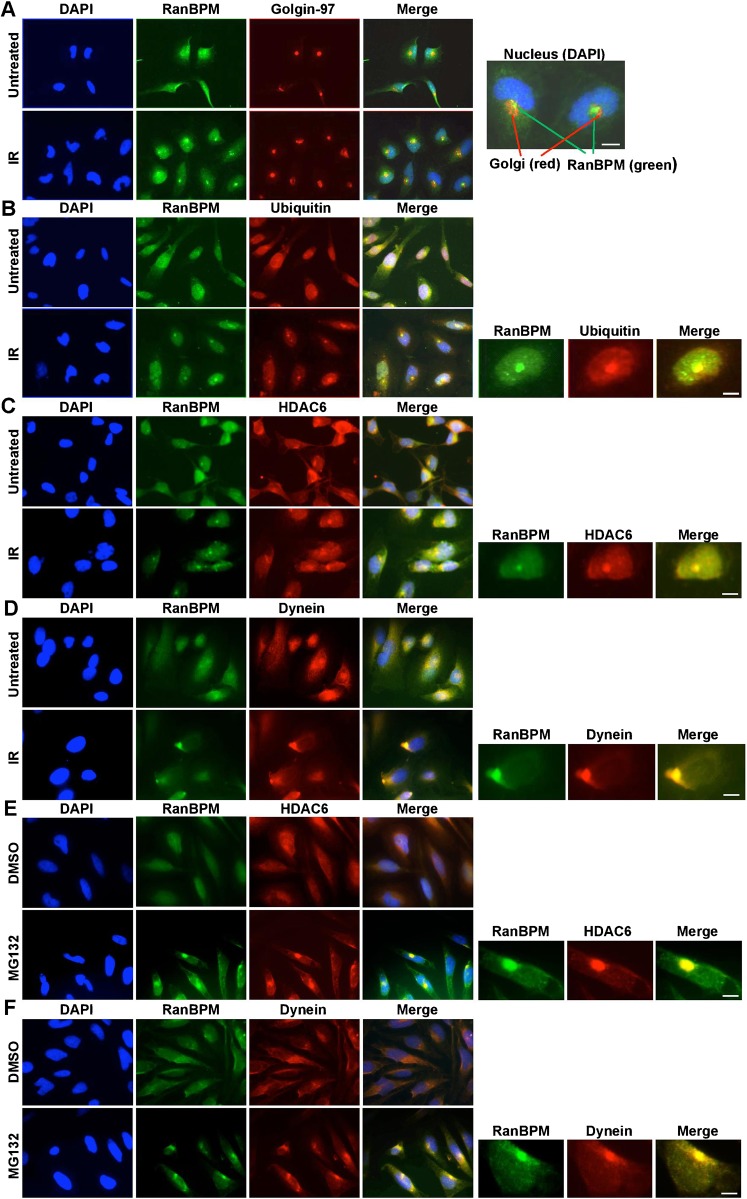
RanBPM is recruited to aggresomes in response to IR and MG132. (A) Hela cells either untreated or 72 h after IR treatment (10 Gy) were immunostained with RanBPM and Golgin-97 antibodies and DAPI. Inset shows close proximity but no colocalization between Golgin-97 and RanBPM. (B) Hela cells were analyzed as described for panel A with RanBPM and ubiquitin antibodies. Inset shows aggresome with colocalization of RanBPM and ubiquitin. (C) Hela cells were analyzed as described above with HDAC6 and RanBPM antibodies. Inset shows aggresome with colocalization of RanBPM and HDAC6. (D) Hela cells were analyzed as described above with dynein and RanBPM antibodies. Inset shows aggresome with colocalization of RanBPM and dynein. (E) Hela cells treated either with DMSO or 10 µM MG132 (16 h) were immunostained with RanBPM and HDAC6 antibodies and DAPI. Inset shows aggresome with colocalization of RanBPM and HDAC6. (F) Hela cells were analyzed as described for panel E with RanBPM and dynein antibodies. Inset shows aggresome with colocalization of RanBPM and dynein. Scale bars: 10 µm.

We previously reported that IR treatment induces RanBPM relocalization from the nucleus to the cytoplasm (Atabakhsh et al., 2009). This relocalization was initiated within 24 hours following IR treatment (10 Gy), but intensified and persisted up to 72 hours. In examining more closely RanBPM staining in Hela cells following IR exposure 72 hours post IR treatment, we noticed that a large proportion of cells displayed perinuclear dots or foci often located close to an invagination of the nucleus. We initially considered the possibility that these foci could be associated with the Golgi apparatus. Co-staining of RanBPM with a Golgi marker (golgin-97) showed a consistent immunostaining of RanBPM foci in the vicinity of the Golgi complex; however, the two signals did not co-localize (Fig. 1A). One particular type of structure that has been reported to localize in the region occupied by the Golgi is the aggresome (Garcia-Mata et al., 2002; Kopito, 2000; Wójcik and DeMartino, 2003). We found that the RanBPM foci formed in our Hela cells bore striking resemblance with previously documented aggresomes in Hela cells (Wójcik et al., 2004). Thus, the general features of these IR-induced RanBPM foci, including their localization with respect to the Golgi apparatus suggested that RanBPM may be recruited to aggresomes in response to IR.

To assess whether RanBPM IR-induced foci were indeed aggresomes, we co-stained Hela cells following IR treatment with an antibody directed against ubiquitin, a well-established marker for aggresomes (Bennett et al., 2005; Kawaguchi et al., 2003; Kopito, 2000; Ouyang et al., 2012; Wójcik et al., 2004). Ubiquitin clearly co-localized to the same aggregates as RanBPM in IR-treated cells (Fig. 1B). To further confirm that these foci were aggresomes, we assessed co-localization of RanBPM with HDAC6 and the molecular motor dynein, both of which are known to be recruited to aggresomes and required for aggresome formation (Boyault et al., 2007b; Johnston et al., 2002; Kawaguchi et al., 2003). Both dynein and HDAC6 were found co-localized with RanBPM in aggresome-like structures (Fig. 1C,D). We obtained similar results in HEK293 cells (data not shown). Altogether, these results suggested that IR treatment triggers the formation of aggresomes, and that RanBPM is recruited to aggresomes.

Since aggresomes have been documented to form in response to proteasome inhibitors, such as MG132, we conducted experiments to determine whether RanBPM was recruited to aggresomes in response to MG132 in Hela cells. Following MG132 treatment, we observed the relocalization of both HDAC6 and dynein with RanBPM to perinuclear aggregates (Fig. 1E,F). While the timeline of aggresome formation was faster in response to proteasomal inhibition (MG132, 16 hours as previously reported (Wójcik et al., 2004)) than in response to IR (72 hours), both treatments triggered morphologically similar structures. Again, we confirmed that this was not a Hela cell-specific response as we also observed RanBPM recruitment to HDAC6-containing aggresomes in HEK293 cells (supplementary material Fig. S1).

### RanBPM down-regulation impairs aggresome formation

**Fig. 2.**
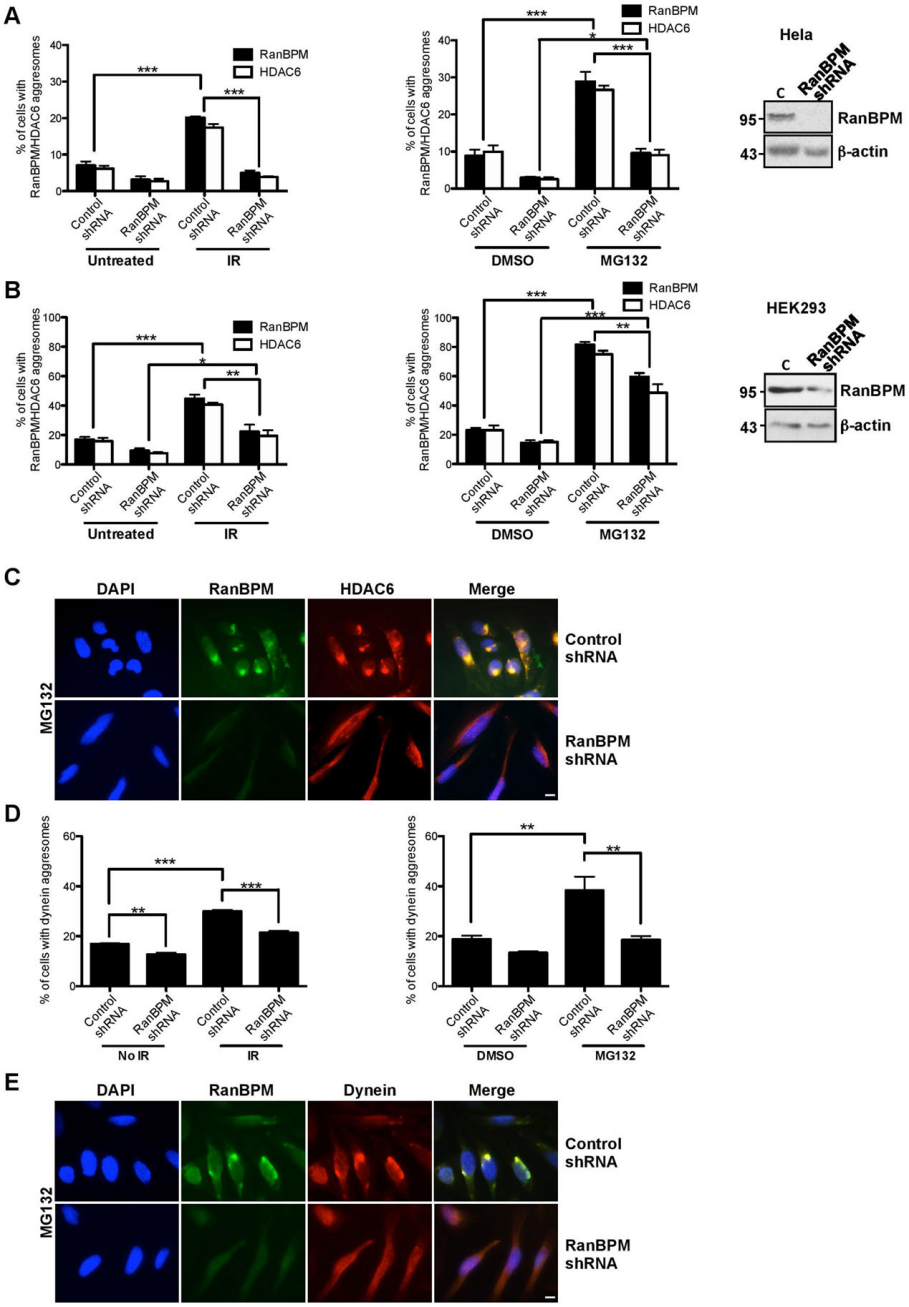
RanBPM downregulation impairs aggresome formation. (A) Hela cells stably expressing control shRNA or RanBPM shRNA were subjected to IR treatment (10 Gy) or left untreated, or treated with MG132 (10 µM) or vehicle (DMSO) and fixed 72 h following IR or 16 h following MG132. Cells were processed for immunostaining with antibodies to RanBPM and HDAC6 and mounted with DAPI. At least 150 cells were scored per experiment for the presence of RanBPM and HDAC6 aggresomes and the results (IR treatment, left graph, and MG132 treatment, right graph) are expressed as percentage of cells containing RanBPM aggresomes (solid bars) or HDAC6 aggresomes (open bars). Results are averaged from three different experiments, with error bars indicating SE. Asterisks indicate statistical significance for the differences in percentage of aggresomes obtained in control versus RanBPM shRNA cells, *P*<0.0005 (***); *P*<0.005 (**); *P*<0.05 (*). Inset, western blot analysis of extracts from Hela control and RanBPM shRNA cells, showing RanBPM expression with respect to a β-actin loading control. (B) HEK293 cells expressing control or RanBPM shRNA were processed and analyzed as described in panel A. Results are expressed as in panel A. Inset, western blot analysis of extracts from HEK293 control and RanBPM shRNA cells, showing the levels of expression of RanBPM versus β-actin used as a loading control. (C) Representative images of Hela cells stably expressing control shRNA or RanBPM shRNA treated with 10 µM MG132 and processed with antibodies to RanBPM and HDAC6 as described in panel A. (D) Hela cells stably expressing control shRNA or RanBPM shRNA were processed with antibodies against RanBPM and dynein and analyzed as described in panel A. The graphs (IR treatment, left graph, and MG132 treatment, right graph) show the percentage of cells containing dynein aggresomes. (E) Representative images of Hela cells stably expressing control shRNA or RanBPM shRNA treated with 10 µM MG132 and processed with antibodies to RanBPM and dynein as described in panel D. Scale bars: 10 µm.

RanBPM expression is not affected either by IR (Atabakhsh et al., 2009) or MG132 treatment (Fig. 6A). In addition, overexpression of RanBPM by transient transfection did not trigger aggresome formation (data not shown). We sought to determine whether RanBPM was simply recruited to aggresomes or if it could play a specific function in the aggresome pathway, in which case its impairment or down-regulation would affect aggresome formation. We previously engineered Hela cells where RanBPM is effectively down-regulated through stable expression of a RanBPM shRNA (Atabakhsh et al., 2009). Thus, we assessed aggresome formation by HDAC6 in RanBPM shRNA Hela cells treated with MG132 or IR. Quantification of aggresome formation in RanBPM shRNA and control shRNA Hela cells revealed that HDAC6 aggresome formation was noticeably impaired in RanBPM shRNA cells both in response to MG132 and IR (Fig. 2A,C). Upon IR treatment, we found that 20.1% of Hela control cells formed RanBPM aggresomes, versus only 4.9% of RanBPM shRNA cells (Fig. 2A). These numbers were similar for HDAC6 (17.4% versus 3.8% respectively). In the conditions used, MG132 treatment (10 µM, 16 hours) was slightly more efficient than IR (10 Gy) at inducing aggresome formation, with 29.0% of control cells displaying RanBPM aggresomes and 26.6% forming HDAC6 aggresomes. MG132 treatment of RanBPM-depleted cells also revealed a drastic reduction of HDAC6 aggresomes (9.5%) compared to control cells (26.6%) (Fig. 2A,C). We obtained similar results in conditions where RanBPM was transiently downregulated via siRNA transfections (supplementary material Fig. S2). To verify that this effect was not cell type-specific, we repeated these experiments in HEK293 cells, in which we previously generated RanBPM shRNA stably expressing cells where RanBPM expression is reduced (Atabakhsh and Schild-Poulter, 2012). Compared to Hela cells, control HEK293 cells displayed a higher percentage of aggresome-bearing cells upon similar MG132 and IR exposure (Fig. 2B; supplementary material Fig. S1). The increased propensity of HEK293 cells to form aggresomes was previously suggested to be due to the expression of the adenoviral protein E1B55K in these cells (Fleisig et al., 2007). In control HEK293 cells, IR treatment induced RanBPM and HDAC6 aggresomes in 44.2% and 40.6% of cells respectively, whereas MG132 treatment triggered aggresome formation in 81.6% (RanBPM) and 75.0% (HDAC6) of control HEK293 cells. Downregulation of RanBPM also significantly reduced aggresome formation by HDAC6 in response to MG132 (48.7%) and IR (19.3%), albeit to a lower extent than in Hela shRNA cells, likely because the HEK293 RanBPM shRNA cells express higher levels of residual RanBPM protein (Fig. 2B, see inset). To ensure that the effect of RanBPM downregulation was not specific to HDAC6, we quantified aggresome formation by dynein in Hela control and RanBPM shRNA cells in response to IR and MG132 (Fig. 2D,E). Similarly to what was observed with HDAC6, aggresome formation by dynein was significantly reduced in MG132-treated Hela RanBPM shRNA cells (18.5%) compared to control cells (38.3%). Likewise, IR treatment triggered 31% of cells to form dynein-containing aggresomes in control cells, whereas only 22% were observed in RanBPM shRNA (Fig. 2D). Altogether, these results suggest that aggresome formation upon MG132 and IR treatment is dependent on RanBPM expression.

### RanBPM is recruited to aggresomes in response to DNA damage

**Fig. 3.**
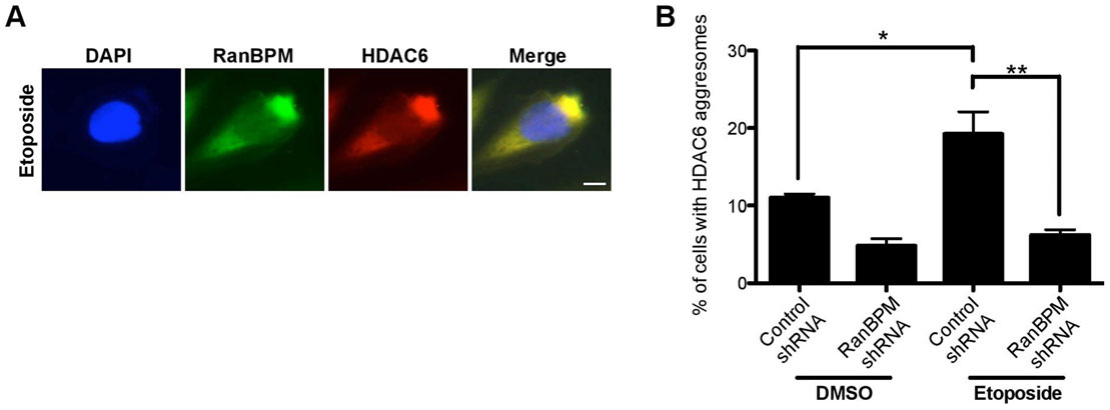
RanBPM localizes to aggresomes with HDAC6 in response to DNA damage. Hela cells stably expressing control shRNA or RanBPM shRNA were fixed 72 h following 1 h treatment with etoposide (2 µM) or vehicle (DMSO). Cells were processed for immunostaining with antibodies to RanBPM and HDAC6 and mounted with DAPI. (A) Representative images of etoposide-treated Hela control cell showing colocalization of RanBPM and HDAC6 in perinuclear aggresome. (B) Quantification of aggresome formation in response to etoposide. At least 100 cells per experiment were scored for HDAC6-containing aggresomes. Results are averaged from three different experiments, with error bars indicating SE. Asterisks indicate statistical significance between treatments and cell lines, *P*<0.005 (**); *P*<0.05 (*). Scale bar: 10 µm.

Since aggresomes are formed in response to UPS defects, we thought it was important to determine whether the induction of aggresomes by IR is triggered specifically through signaling by the DNA damage response to the UPS, or is due to secondary effects of IR, such as protein oxidation. We used Hela cells to assess aggresome formation by RanBPM and HDAC6 in response to etoposide, which specifically causes double-stranded breaks (DSBs) through inhibition of topoisomerase II (Cummings and Smyth, 1993). We treated cells with 2 µM etoposide, a dose previously shown to instigate comparable DNA damage as 10 Gy of IR (Pérez et al., 1997) and processed samples for HDAC6 and RanBPM immunofluorescence 72 hours following treatment. Similarly to IR, etoposide treatment triggered the formation of co-localized RanBPM and HDAC6 perinuclear aggregates (Fig. 3A). Quantifications indicated that 19.1% of control cells displayed HDAC6 aggresomes (Fig. 3B), which closely matched the number obtained in response to IR (17.4%, Fig. 2A) and this number was significantly reduced in RanBPM shRNA cells (6.5%). Quantification of RanBPM aggresomes yielded similar numbers (data not shown). This confirmed that DSBs can elicit a response leading to the formation of aggresomes.

### RanBPM forms a complex with HDAC6

**Fig. 4.**
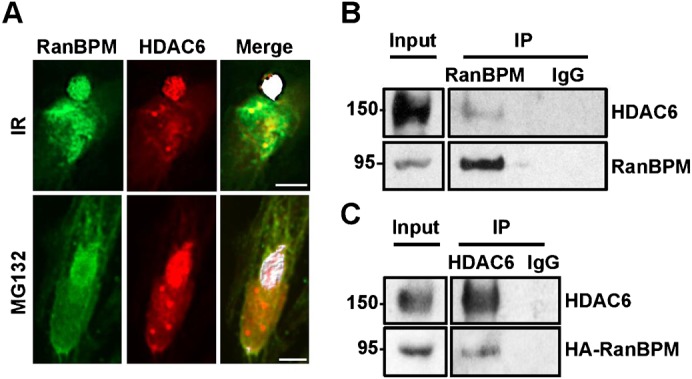
RanBPM forms a complex with HDAC6. (A) IR (10 Gy, 72 h) and MG132 (10 µM, 16 h) treated Hela cells were analyzed using confocal microscopy, and colocalization of HDAC6 and RanBPM analyzed using Imaris software. White signal represents RanBPM and HDAC6 colocalization. (B) Control shRNA Hela whole cell extracts were incubated with either a RanBPM antibody or mouse IgG control. Immunoprecipitates were analyzed by western blot using HDAC6 and RanBPM antibodies and compared with 5% of input proteins. (C) Whole cell extracts of RanBPM shRNA Hela cells transfected with pCMV-HA-RanBPM si-mt were incubated with an HDAC6 antibody or mouse IgG control. Immunoprecipitates were analyzed by western blot with HA and HDAC6 antibodies and compared to 5% input extracts. Scale bars: 10 µm.

Confocal microscopy analyses conducted on both IR and MG132-treated cells revealed that RanBPM and HDAC6 co-localized within the aggresome (as evidence by the white co-localized voxels, Fig. 4A), suggesting that the two proteins interact. To corroborate these findings, we performed co-immunoprecipitation analysis to determine a potential association of RanBPM with HDAC6. Endogenous HDAC6 was indeed co-immunoprecipitated with endogenous RanBPM in untreated Hela cells, suggesting that the two proteins form a complex (Fig. 4B). Reversely, immunoprecipitation of endogenous HDAC6 was found to co-immunoprecipitate HA-RanBPM expressed in Hela RanBPM shRNA cells, which confirmed complex formation between RanBPM and HDAC6 (Fig. 4C). Altogether, these analyses suggest that RanBPM associates with HDAC6 both in untreated cells and within aggresome structures.

### RanBPM expression inhibits HDAC6 activity

**Fig. 5.**
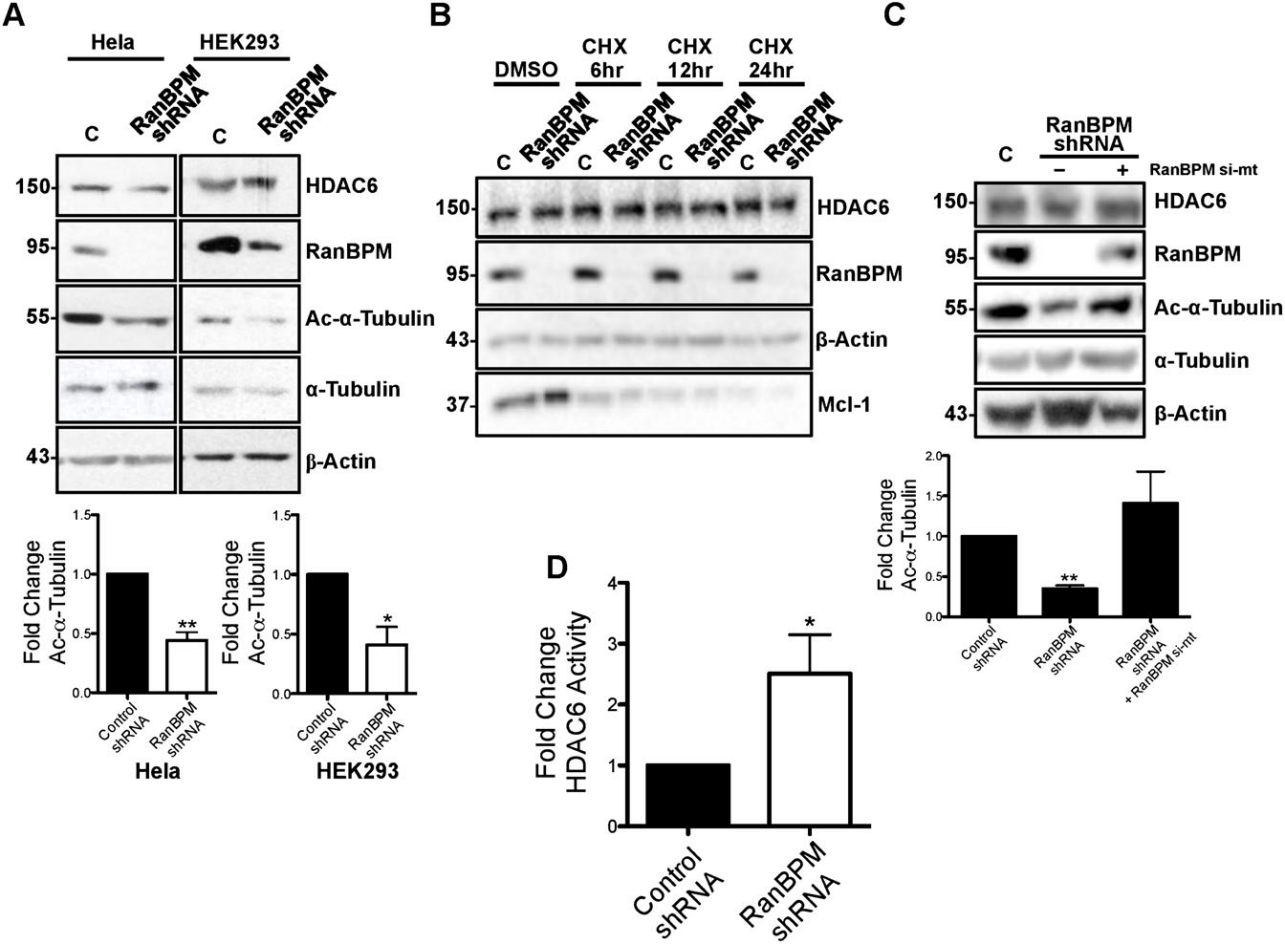
RanBPM inhibits HDAC6 activity. (A) *Top*, Hela and HEK293 control shRNA (C) and RanBPM shRNA whole cell extracts were analyzed by western blotting and hybridized with the indicated antibodies. *Bottom*, quantification of relative amounts of acetylated α-tubulin was normalized to total α-tubulin levels. Results are averaged from three different experiments, with error bars indicating SD. *P*<0.005 (**); *P*<0.05 (*). (B) Control shRNA (C) and RanBPM shRNA Hela cells were treated with either DMSO or 25 µg/ml CHX, for the times indicated and whole cell extracts were analyzed by western blotting and hybridized with the antibodies indicated. (C) *Top*, Control shRNA (C) and RanBPM shRNA Hela cells were left untransfected (–) or transfected with pCMV-HA-RanBPM si-mt and whole cell extracts were prepared and analyzed by western blotting and hybridized with the antibodies indicated. *Bottom*, quantification of relative amounts of acetylated α-tubulin was normalized to total α-tubulin levels. Results are averaged from three different experiments, with error bars indicating SD. *P*<0.005 (**). (D) The activity of HDAC6 immunoprecipitates from Hela control shRNA and RanBPM shRNA was measured using a deacetylation assay. Shown is the RanBPM shRNA HDAC6 immunoprecipitate activity normalized that of control shRNA. Results are averaged from three different experiments, with error bars indicating SD. *P*<0.05 (*).

To start investigating the effect of RanBPM interaction with HDAC6, we first determined whether RanBPM expression affected HDAC6 protein levels and activity towards its substrate α-tubulin. A comparison of HDAC6 protein levels in Hela and HEK293 cells expressing control or RanBPM shRNA did not reveal any obvious effect of RanBPM on HDAC6 protein expression (Fig. 5A). In addition, treatment with the protein synthesis inhibitor cycloheximide (CHX) revealed no change and no difference in HDAC6 levels between RanBPM shRNA cells and control cells, inferring that RanBPM does not affect HDAC6 protein stability (Fig. 5B). However, the levels of acetylated α-tubulin were found significantly decreased in RanBPM shRNA cells, suggesting that RanBPM downregulation resulted in enhanced HDAC6 deacetylase activity (Fig. 5A). Quantification indicated at least 2-fold reduction of acetylated α-tubulin in RanBPM shRNA cells compared to control cells (Fig. 5A, lower panel). Re-expression of RanBPM in RanBPM shRNA cells restored α-tubulin acetylation, thus confirming that this effect was specific to RanBPM expression (Fig. 5C). To substantiate that α-tubulin decrease in acetylation was due to an increase in HDAC6 deacetylase activity in RanBPM shRNA cells, we conducted deacetylation assays using a fluorometric HDAC activity assay. We compared deacetylase activity of HDAC6 immunoprecipitates prepared from control and RanBPM shRNA cells. RanBPM shRNA cells showed a 2.5 fold higher HDAC6 activity compared to control cells, indicating that the downregulation of RanBPM increases HDAC6 activity (Fig. 5D). Altogether, these experiments suggest that RanBPM exerts an inhibitory effect on HDAC6 activity.

**Fig. 6.**
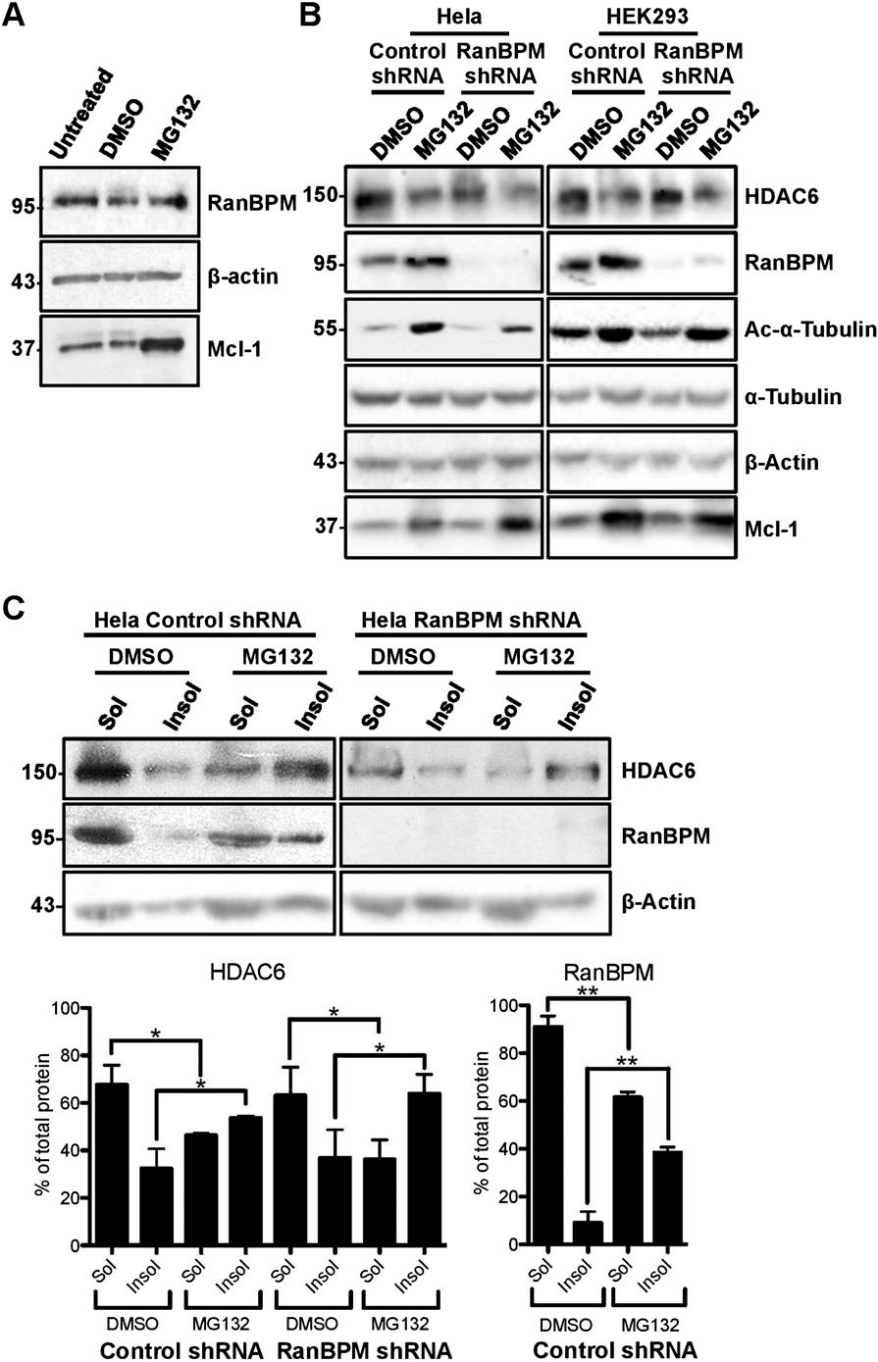
Effect of RanBPM expression and MG132 treatment on tubulin acetylation and HDAC6 levels and solubility. (A) Control shRNA Hela whole cell extracts either untreated or treated with DMSO or 10 µM MG132 for 16 h were analyzed by western blotting and hybridized with the antibodies indicated. (B) Control shRNA and RanBPM shRNA Hela and HEK293 cells were treated with either DMSO or 10 µM or 5 µM MG132, respectively for 16 h and whole cell extracts were analyzed by western blotting and hybridized with the antibodies indicated. (C) *Top*, Hela control shRNA or RanBPM shRNA cells were treated with either DMSO or 10 µM MG132 for 16 h were fractionated into soluble (Sol) or insoluble (Insol) fractions and analyzed by western blotting with antibodies to HDAC6, RanBPM and β-actin. *Bottom*, quantification of HDAC6, and RanBPM in soluble and insoluble fractions. Graphs show the percentage of protein present in each fraction for each treatment condition. Results are averaged from three different experiments, with error bars indicating SD. *P*<0.005 (**); *P*<0.05 (*).

We then assessed a potential effect of RanBPM on HDAC6 levels and activity following proteasome inhibition by MG132. RanBPM protein levels were unaffected upon MG132 treatment (Fig. 6A), whereas those of the anti-apoptotic factor Mcl-1 used as a control increased significantly, as expected (Yuan et al., 2008). By contrast, HDAC6 protein levels were surprisingly found to decline in conditions of proteasomal impairment (Fig. 6B). Previous studies found that MG132 treatment results in the redistribution of HDAC6 (and other aggresome proteins) into insoluble fractions (Fusco et al., 2012; Guthrie and Kraemer, 2011; Zucchelli et al., 2009), so we reasoned that the MG132-dependent decrease in HDAC6 protein levels detected by analysis of whole cell extracts may be reflecting an increase in detergent-insoluble HDAC6. Thus, we tested the effect of RanBPM expression on the distribution of HDAC6 in soluble and insoluble fractions following MG132 treatment (Fig. 6C). In untreated cells, 68% of HDAC6 was present in the soluble fraction, versus 32% in the insoluble fraction. However, this distribution was reversed in MG132-treated cells, with 53% of HDAC6 found in the insoluble fraction. In the same fractions, RanBPM was mostly soluble (91%) in untreated cells, but MG132 treatment caused a significant accumulation (38%) in the insoluble fraction. However, we did not detect any significant change in HDAC6 redistribution in insoluble versus soluble fractions in RanBPM shRNA cells in response to MG132 compared to control cells, suggesting that RanBPM expression does not affect HDAC6 solubility. Thus, RanBPM does not appear to affect HDAC6 expression or solubility in normal and MG132-treated cells.

To identify a potential effect of RanBPM on HDAC6 activity in conditions of proteasome inhibition, we analyzed acetylated α-tubulin in whole cell extracts (Fig. 6B). MG132 treatment elicited a marked increase in acetylated α-tubulin in both Hela and HEK293 control cells. In RanBPM down-regulated cells, MG132 treatment also triggered an increase in acetylated α-tubulin. While this increase appeared slightly dampened by the lack of RanBPM, quantifications did not reveal this decrease to be significant (data not shown) suggesting that MG132 effect on α-tubulin acetylation is independent of RanBPM expression. Altogether, these results suggest that RanBPM inhibits HDAC6 activity and that proteasome inhibition affects HDAC6 solubility and α-tubulin acetylation through mechanisms that appear to be independent of RanBPM expression.

### Deletion of the RanBPM LisH/CTLH domain prevents HDAC6 interaction and aggresome formation

**Fig. 7.**
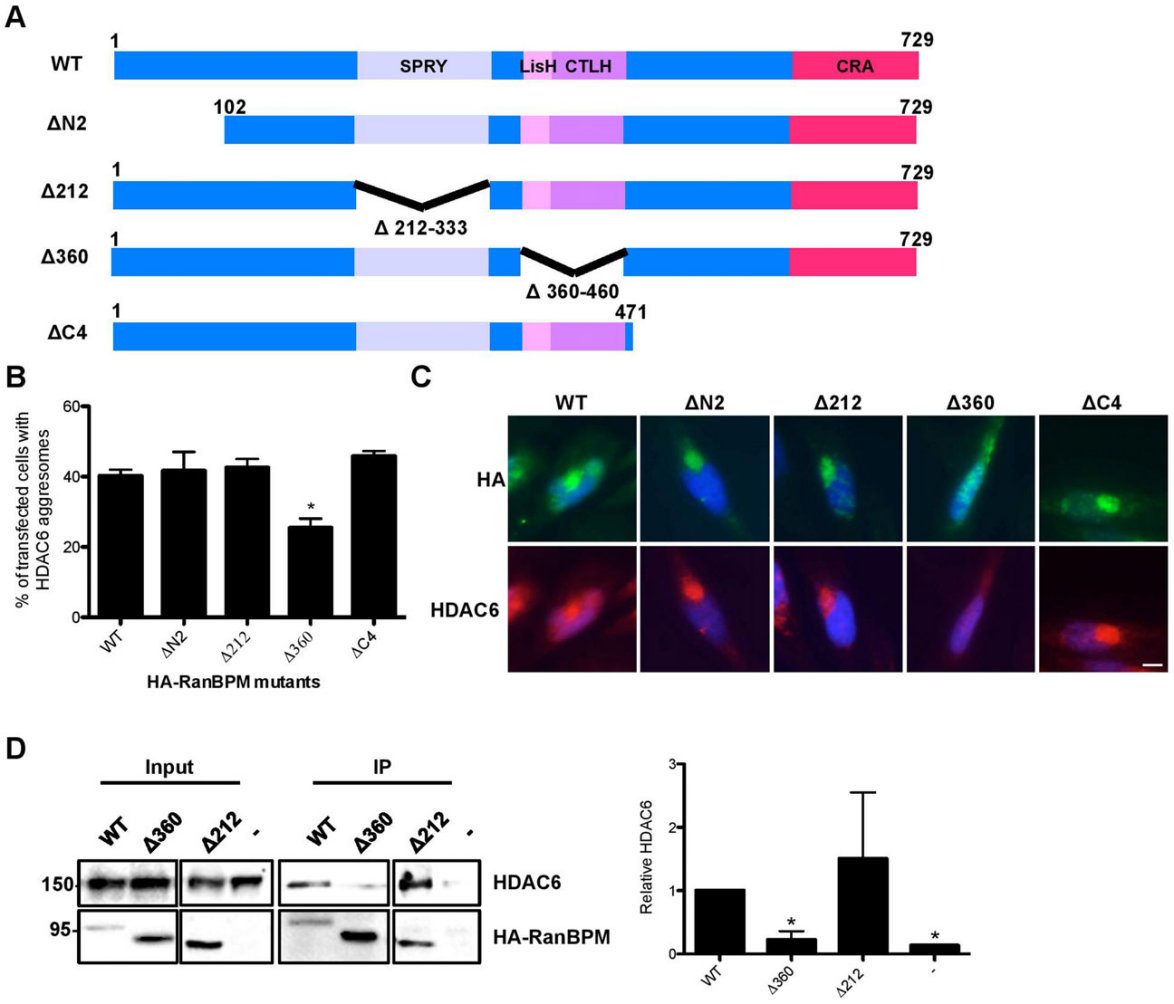
The RanBPM LisH/CTLH domain is required for aggresome formation and HDAC6 interaction. (A) Schematic representations of HA-tagged RanBPM wildtype (WT) and deletion mutant constructs ΔN2, Δ212, Δ360 and ΔC4. The RanBPM conserved domains are indicated (CRA, CT11-RanBPM). (B) Hela cells stably expressing RanBPM shRNA were transfected with HA-WT, HA-ΔN2, HA-Δ212, HA-Δ360 and HA-ΔC4 and were fixed 16 h following 10 µM MG132 treatment. Cells were processed for immunostaining with antibodies to HA and HDAC6 and mounted with DAPI. At least 100 transfected cells were scored per experiment for the presence of aggresomes and the results are expressed as percentage of cells containing HDAC6 aggresomes. Results are averaged from four different experiments, with error bars indicating SE. *P*<0.05 (*). (C) Representative images of HA-tagged RanBPM constructs transfected into RanBPM shRNA Hela cells and treated with 10 µM MG132, processed as described above. (D) The RanBPM LisH/CTLH domain is necessary for interaction with HDAC6. *Right*, whole cell extracts were prepared from RanBPM shRNA Hela cells untransfected (–) or transfected with HA-WT-, HA-Δ360 or HA-Δ212 constructs. RanBPM was immunoprecipitated with a RanBPM antibody, and immunoprecipitates analyzed by western blot with an HDAC6 antibody. RanBPM WT and deletion mutant immunoprecipitation was verified using an HA antibody. Input, 5% input extract. *Left*, quantification of relative amounts of co-immunoprecipitated HDAC6 normalized to immunoprecipitated RanBPM. Results are averaged from three different experiments with error bars indicating SD. *P*<0.05 (*). Scale bar: 10 µm.

Since RanBPM was found to form a complex with HDAC6 and was required for aggresome formation, we investigated whether the interaction of RanBPM with HDAC6 was a prerequisite for aggresome formation. HA-RanBPM wild-type (WT) or deletion mutants lacking either the N-terminus (ΔN2), the C-terminus (ΔC4), the SPRY domain (Δ212) or the LisH/CTLH domain (Δ360) (Fig. 7A) were transiently expressed in Hela RanBPM shRNA cells, and assessed for their ability to form aggresomes in response to MG132 treatment. All HA-RanBPM constructs contain a point mutation in the sequence targeted by the RanBPM siRNA (except for ΔC4 which lacks the targeted sequence), as previously described (Atabakhsh et al., 2009). Aggresome formation by HA-RanBPM WT and mutants was assessed by quantifying the number of aggresomes where transfected RanBPM was found to co-localize with endogenous HDAC6 (Fig. 7B,C). Re-introduction of WT RanBPM re-established aggresome formation, as 38% of transfected cells showed aggresomes positive for HDAC6 and RanBPM, similar to that found in control Hela cells (Fig. 2A). Similarly, RanBPM mutants ΔN2, ΔC4 and Δ212 were able to rescue aggresome formation to levels comparable to WT RanBPM. However, the RanBPM Δ360 mutant was unable to induce aggresome formation to the expected level, suggesting that the LisH/CTLH domain is involved in mediating aggresome formation. To determine whether this domain was involved in complex formation with HDAC6, we performed immunoprecipitations of HA-RanBPM WT, Δ360 and Δ212 and assessed endogenous HDAC6 association. Co-immunoprecipitation of HDAC6 with RanBPM Δ360 was markedly reduced compared to RanBPM WT and Δ212, suggesting that deletion of the LisH/CTLH domain severely impairs the interaction of RanBPM with HDAC6 (Fig. 7D). These results indicate that the RanBPM LisH/CTLH domain is involved in both HDAC6 interaction and aggresome formation, suggesting that complex formation of HDAC6 with RanBPM is involved in regulating aggresome formation.

## DISCUSSION

The regulatory mechanisms underlying aggresome formation and the key proteins involved in this process remain poorly understood. Here we report that aggresome formation can be elicited by exposure to DNA damaging agents and that the protein RanBPM plays an essential role in the aggresome pathway. We present evidence that RanBPM forms a complex with HDAC6 and inhibits HDAC6 activity and that RanBPM function in aggresome formation is dependent on its association with HDAC6.

HDAC6 is a critical regulator of aggresome formation and cells deficient in HDAC6 cannot form aggresomes (Kawaguchi et al., 2003). HDAC6 interacts with the microtubule-associated motor dynein and with polyubiquitinated misfolded proteins, functioning to recruit protein cargo to dynein motors for transport to the aggresome (Kawaguchi et al., 2003). We have shown here that RanBPM associates with HDAC6 and that downregulation of RanBPM results in a strong reduction in aggresome formation, suggesting that the lack of RanBPM causes a severe disruption in the aggresome pathway. Expression of a RanBPM mutant (Δ360) that impaired complex formation with HDAC6 elicited a modest, but significant reduction in aggresome formation by HDAC6. This suggests that the association of RanBPM with HDAC6 that is observed in the absence of stress promotes HDAC6 function in the aggresome pathway in conditions of proteasome impairment. Yet, whether this interaction is direct or mediated by other proteins remains to be determined. Interestingly, the RanBPM region identified as interacting with HDAC6 is a LisH/CTLH domain, which is found in proteins that interact with microtubules (Emes and Ponting, 2001). Functional studies of LisH motif-containing proteins suggest that LisH motifs mediate microtubule binding and/or metabolism (Emes and Ponting, 2001). For instance, Lissencephaly (LIS1), the best characterized LisH-containing protein, functions in microtubule organization and homeostasis through binding to dynein and regulation of dynein motor function (Yamada et al., 2008). The LisH motif of LIS1 is not involved in dynein binding, but mediates LIS1 dimerization, which is essential for its regulatory function of dynein motility (Torisawa et al., 2011). HDAC6 interacts with microtubules and dynein and also co-localizes with p150^glued^, a subunit of the dynactin complex (Hubbert et al., 2002; Kawaguchi et al., 2003). Thus RanBPM association with HDAC6 could be functioning to regulate HDAC6 function in microtubule-based cargo transport to the aggresome. It was previously suggested that RanBPM interacted with microtubules, but this observation was later dismissed as the original study used an antibody that did not recognize RanBPM (Nakamura et al., 1998; Nishitani et al., 2001). Some studies have subsequently suggested a potential role for RanBPM in microtubule regulation (Menon et al., 2004; Togashi et al., 2006), although a direct association of RanBPM with microtubules remains to be confirmed. Interestingly, the highly similar protein RanBP10, whose expression is restricted to hematopoietic cell lineages, has been shown to function in platelet microtubule organization through an interaction with β1-tubulin (Kunert et al., 2009; Schulze et al., 2008). RanBPM and RanBP10 display 67% amino acid sequence identity and, while having divergent N-terminal domains, share a SPRY, LisH, and CTLH domains (Deshmukh and Johnson, 1998; Wang et al., 2004). Thus, microtubule association and regulation may be a common feature of both proteins; however, their functions at microtubules appear to be distinct since the RanBP10 knockout mouse platelet microtubule defects are not compensated for by RanBPM (Kunert et al., 2009; Meyer et al., 2012). Interestingly, HDAC6 was recently shown to regulate tubulin deacetylation during platelet activation, raising the intriguing possibility of a potential interplay between HDAC6 and RanBP10 in platelet activation (Sadoul et al., 2012).

In addition to HDAC6, several proteins have been shown to regulate aggresome formation. Several of these factors are either chaperones or part of ubiquitin/deubiquitin complexes, such as Hsp70, the ubiquitin ligase CHIP and the deubiquitinating enzyme AT3 (Chin et al., 2008; Sha et al., 2009). In yeast, RanBPM has been found to be associated with a multi-subunit ubiquitin ligase complex called the Vid or Gid complex (Menssen et al., 2012). In mammalian cells RanBPM has been shown to be part of large cytosolic complex called the CTLH complex (Kobayashi et al., 2007; Umeda et al., 2003). The mammalian homologs of several Gid/Vid proteins have been found to be part of the CTLH complex and/or interact with RanBPM, suggesting that the CTLH complex may be the mammalian ortholog of the Gid/Vid complex (Kobayashi et al., 2007; Menssen et al., 2012; Umeda et al., 2003). This raises the possibility that RanBPM may be functioning in the aggresome pathway as part of this complex, but whether it is associated with ubiquitin ligase activity remains to be demonstrated.

We found RanBPM to have a negative effect on HDAC6 activity. The levels of acetylated α-tubulin, a known substrate of HDAC6 were found reduced in both Hela and HEK293 RanBPM shRNA cells. Using a deacetylation assay, we detected an increased deacetylation activity in RanBPM Hela shRNA cells, further suggesting that RanBPM functions as an HDAC6 inhibitor. Interestingly, two other HDAC6 inhibitors identified so far, Tau and TPPP/p25, are microtubule-interacting proteins that are recruited to aggresomes in response to proteasome inhibition (Guthrie and Kraemer, 2011; Lehotzky et al., 2004; Perez et al., 2009; Toőkési et al., 2010). How these two proteins inhibit HDAC6 remains to be detailed, but this suggests that HDAC6 activity at microtubules is subjected to multiple regulations.

It is unclear at present whether the inhibitory effect of RanBPM on HDAC6 deacetylase activity is linked to its function in aggresome formation. HDAC6 deacetylase activity has been demonstrated to be essential for aggresome formation (Kawaguchi et al., 2003; Watabe and Nakaki, 2011). Thus, relieving an inhibitory effect on HDAC6 deacetylase activity through RanBPM downregulation would have been expected to facilitate aggresome formation; however, the opposite effect was observed. On the other hand, the possibility exists that HDAC6 hyperactivity resulting from RanBPM downregulation could be detrimental to aggresome formation. Notwithstanding, the regulation of HDAC6 deacetylase activity may not be the mechanism through which RanBPM functions to regulate aggresome formation. In support to this, we observed increased α-tubulin acetylation upon MG132 treatment (and in response to IR, data not shown) and this occurred in both control and RanBPM shRNA cells and thus appeared to be independent of RanBPM. Hyperacetylation of α-tubulin in response to proteasome inhibitors was previously noted by others; however, the mechanism by which this hyperacetylation occurs has not been elucidated (Diaz-Corrales et al., 2012; Poruchynsky et al., 2008). Tubulin acetylation has been linked to increased microtubule transport and was shown to promote the recruitment of dynein to microtubules (Dompierre et al., 2007). Consistent with a previous report (Fusco et al., 2012), our results show that HDAC6 insolubility is increased upon MG132 treatment; however, this was not affected by RanBPM either. This effect could also be linked to tubulin hyperacetylation, since increased acetylation of tubulin has been linked to its insolubility (Scharadin et al., 2012; Zuccotti et al., 2012). Thus, tubulin acetylation may be a prerequisite for transport of cargo to the aggresome but RanBPM does not appear to be involved in this regulation in conditions of proteasome impairment. Hence, the role of RanBPM in aggresome formation may be to promote the processivity of HDAC6 along microtubules but how this is achieved remains to be determined.

The inhibitory role of RanBPM on HDAC6 activity could nonetheless have important consequences on other cellular processes. Increased HDAC6 activity is known to be associated with increased cell motility, in part through deacetylation of α-tubulin, but also due to the increased chaperone function of deacetylated Hsp90 towards oncogenic proteins such as Akt/PKB, ErbB2 and c-Raf (Aldana-Masangkay and Sakamoto, 2011; Mollapour and Neckers, 2012). Increased HDAC6 levels have been noted in certain tumor types, such as oral squamous cell cancer, ovarian cancer and glioma (Aldana-Masangkay and Sakamoto, 2011; Witt et al., 2009; Wu et al., 2010). Interestingly, our previous studies showed that RanBPM expression inhibits the ERK pathway through a regulation of c-Raf stability and also restricts cell proliferation and mobility (Atabakhsh and Schild-Poulter, 2012). We previously attributed the effect of RanBPM on cell motility to its negative regulation of the ERK pathway; however, in light of the results of this study, it is possible that inhibition of HDAC6 activity could account, at least in part, for the inhibitory effect of RanBPM on cell migration.

To our knowledge, this is the first report documenting aggresome formation in response to IR or other DNA damaging agents. We have shown here that both IR and etoposide that specifically induce DSBs result in the formation of aggresomes. Intriguingly, however, we only observed aggresomes in response to high doses of IR (10 Gy) which induce massive cell death (Atabakhsh et al., 2009). Also, aggresome formation did not occur as an immediate response to the DNA injury but appeared to be a delayed consequence of the DNA damage. Aggresomes were first noticed around 60 hours following IR treatment (data not shown), and their accumulation appeared maximal at 72 hours following IR treatment. We previously determined that this correlates with the onset of apoptosis in these cells (Atabakhsh et al., 2009), suggesting a link between aggresome formation and apoptosis. Previous studies have reported that the proteasome is inhibited through caspase-mediated cleavage following the activation of apoptosis in response to various apoptotic stimuli, including DNA damage (Sun et al., 2004). The inactivation of the proteasome after the initiation of apoptosis was suggested to facilitate and amplify the apoptotic cascade (Friedman and Xue, 2004; Sun et al., 2004). Therefore, aggresome formation may be a consequence of the loss of proteasome function in the early stages of apoptosis. In RanBPM shRNA cells, decreased aggresome formation could therefore be the combined result of reduced apoptosis activation and impaired aggresome formation, both of which occurring due to the loss of RanBPM.

This study has uncovered a new role for RanBPM in aggresome formation and as an HDAC6 inhibitor. These findings have important consequences for cellular processes related to cancer and neurodegenerative pathologies, in which both RanBPM and HDAC6 have previously been implicated. HDAC6 overexpression has been linked to cancer development in several tissues, cancer cell lines and tumour mouse models (Witt et al., 2009). Conversely, several studies, including ours, have suggested that RanBPM has tumour suppressor functions by promoting apoptosis and inhibiting cell proliferation and migration (Atabakhsh et al., 2009; Atabakhsh and Schild-Poulter, 2012; Kramer et al., 2005; Suresh et al., 2010). Thus, tumour suppressive functions of RanBPM may be at least in part linked to its ability to repress the oncogenic effects of HDAC6 activity. HDAC6 has also been implicated as a key player in axonal transport and protein aggregation in several neurodegenerative processes including Alzheimer's disease (AD), Parkinson's disease (PD) and amyotrophic lateral sclerosis (ALS) (d'Ydewalle et al., 2012; Simões-Pires et al., 2013). In turn, RanBPM has been implicated in the pathogenesis of AD, in part through the potentiation of amyloid-β peptide generation (Lakshmana et al., 2010; Lakshmana et al., 2009). Thus the interplay between RanBPM and HDAC6 that we have uncovered in this study may also help understand the cellular pathology underlying protein aggregation in neurodegenerative diseases.

## MATERIALS AND METHODS

### Plasmids expression constructs

pCMV-HA-RanBPM shRNA mutant construct (HA-RanBPM si-mt) and pCMV-HA-RanBPM-ΔN2 (ΔN2) were previously described (Atabakhsh et al., 2009). pCMV-HA-RanBPM-Δ212 (Δ212) and pCMV-HA-RanBPM-Δ360 (Δ360) mutant constructs were generated in pCMV-HA-RanBPM shRNA si-mt using inverse polymerase chain reaction (PCR) using tail-to-tail primers on each side of the region to be deleted. pCMV-HA-RanBPM-ΔC4 (ΔC4) was generated by PCR amplification of RanBPM (aa 1–471) and cloning into pCMV-HA digested with XhoI and SalI. PCR reactions were done using PfuTurbo from Agilent Technologies (Mississauga, ON, Canada) and primers from Sigma–Aldrich (Oakville, ON, Canada).

### Cell culture and treatments

Hela and HEK293 control shRNA and RanBPM shRNA stable cell lines were described previously (Atabakhsh et al., 2009; Atabakhsh and Schild-Poulter, 2012) and were cultured in high glucose Dulbecco's modified Eagle's medium (DMEM) supplemented with 10% fetal bovine serum (FBS) and 0.35 mg/ml G418 (Hela) or 0.45 mg/ml G418 (HEK293) (Geneticin, Bioshop Canada, Burlington, ON, Canada) at 37°C in 5% CO_2_. Ionizing radiation (IR) treatments (10 Gy) were performed with a Faxitron RX-650 at a dose rate of 1.42 Gy/min on cells plated the night before irradiation at 50–60% confluency. For MG132 treatment, Hela and HEK293 cells were incubated with 10 µM or 5 µM MG132 (EMD-CalBiochem, San Diego, CA) respectively, for 16 hours. For etoposide treatment, cells were incubated in medium containing 2 µM etoposide (Sigma–Aldrich) for 1 hour, washed and changed to regular medium and incubated for 72 hours before analysis. Cycloheximide (CHX) treatments were performed by incubating cells in medium containing 25 µg/ml CHX (Sigma–Aldrich) for the indicated times (6–24 hours).

### Transfection assays

Plasmid transfections were carried out with TurboFect Transfection Reagent (Thermo Fischer Scientific, Burlington, ON, Canada) according to the manufacturer's protocol. siRNA transfections were carried out as previously described (Atabakhsh et al., 2009; Boyault et al., 2007a).

### Extract preparation, western blot and immunoprecipitations

Whole cell extracts were prepared as described (Atabakhsh et al., 2009) and resolved by SDS-PAGE (between 8% and 12%). Preparation of soluble/insoluble fractions was adapted from Garcia-Mata et al. (García-Mata et al., 1999). Briefly, cells were lysed in RIPA buffer (50 mM Tris-HCl, pH 8, 1% NP-40, 0.05% deoxycholate, 0.1% SDS, and 150 mM NaCl) supplemented with protease inhibitors. Lysates were then passed 10 times through a 25-gauge needle. Insoluble material was collected after centrifugation and pellets were resuspended in 1% SDS in PBS. Equal volumes of pellets and supernatants were resolved by SDS-PAGE (between 8% and 12%). For western blot analyses, gels were transferred on PVDF membranes and hybridized with the following antibodies: RanBPM (5M, Bioacademia, Japan), β-actin (I-19, Santa Cruz, Santa Cruz, CA, USA), HA (HA-7, Sigma–Aldrich), HDAC6 (D-11 and H-300, Santa Cruz), acetylated α-tubulin (6-11B-1, Santa Cruz), α-tubulin (Sigma–Aldrich), Mcl-1 (S-19, Santa Cruz). The blots were developed using the Western Lightning® Enhanced Chemiluminescence Reagent (Perkin Elmer, Waltham, MA, USA). Quantifications were done using ImageJ software and Image Lab (BioRad, Hercules, CA).

For co-immunoprecipitation experiments, extracts were adjusted to 0.25% NP-40 and 100 mM KCl, immunoprecipitations were carried out overnight at 4°C with antibodies to RanBPM (F-1, Santa Cruz) or HDAC6 (D-11, Santa Cruz). Immunoprecipitates were isolated with Dynabeads® protein G (Invitrogen, Life Technologies, Burlington, ON, Canada).

### Immunofluorescence

Cells were plated on coverslips and incubated overnight and treated as described in figure legends. Cells were fixed with 3% paraformaldehyde, permeabilized in 0.5% Triton X-100 for 10 minutes and pre-blocked in 5% FBS diluted in PBS. Coverslips were incubated overnight with primary antibodies (see below), washed in PBS and incubated with secondary antibodies: anti-rabbit Alexa Fluor 488, anti-goat Alexa Fluor 488 and anti-mouse Alexa Flour 488, anti-goat Alexa Fluor 594, anti-rabbit and anti-mouse Alexa Fluor 647 (Invitrogen). Cells were mounted with ProLong® Gold antifade with DAPI (Invitrogen). Visualization was done with an Olympus BX51 microscope with a 40× objective and images were captured with the Image-Pro Plus software (Media Cybernetics Inc., Bethesda, MD, USA). Primary antibodies used in immunofluorescence: RanBPM (Ab5295, Abcam and K-12, Santa Cruz), HA (HA-7, Sigma–Aldrich), HDAC6 (H-300, Santa Cruz), ubiquitin (Sigma–Aldrich), dynein (clone 70.1, Sigma–Aldrich.) and Golgin-97 (Thermo Scientific). For quantification analysis, images were blinded by a third party and coded images were scored independently by two individuals. Aggresomes were scored on the criteria of size (min. 1 µm), signal intensity and perinuclear localization. For each treatment, at least 100 cells per sample were scored by each individual and results were averaged from at least three separate experiments. Confocal images were acquired using an inverted IX51 Olympus microscope equipped with a Perkin Elmer Spinning Disk Confocal attachment with a 60× objective. Image deconvolution was done with AutoQuant software (AutoQuant Imaging, Burnbury, Ontario, Canada) and co-localization analyses were done using Imaris software (Bitplane, Zurich, Switzerland).

### HDAC assay

HDAC6 was immunoprecipitated as described above and immunoprecipitates were resuspended in assay buffer (1 mM KCl, 10 mM HEPES (pH 7.4), 1.5 mM MgCl2, 1 mM DTT, 1 mM phenylmethylsulfonyl fluoride, 5 µg/mL leupeptin 2 µg/mL aprotinin and 10% glycerol). HDAC activity was measured using a HDAC Fluorometric Activity Assay Kit (Cayman Chemical, Ann Arbor, MI, USA) according to manufacturer's protocol. Briefly, immunoprecipitates with and without HDAC inhibitor (1 µM TSA) in duplicate wells, were incubated with an HDAC substrate (200 µM). Deacetylated substrate was measured at 450 nm using a SpectraMax M5 fluorimeter. Average fluorescence of TSA treated samples was subtracted from the average of untreated corresponding samples. HDAC Activity was determined using the deacetylated product concentration obtained using the deacetylated standard curve. HDAC activity is represented as fold activation of HDAC activity in Hela RanBPM shRNA extracts normalized to Hela control shRNA extracts.

### Statistical analyses

Differences between two groups were compared using unpaired two-tailed t test and analysis of variance (ANOVA) was used when comparing multiple groups. Results were considered significant when *P*<0.05.

## Supplementary Material

Supplementary Material
