# Supplementary material for: Aggresome formation is regulated by RanBPM through an interaction with HDAC6
Source: Biol Open. 2014 May 2;3(6):418–30. doi: 10.1242/bio.20147021 (PMC4058076; doi:10.1242/bio.20147021)
Supplement: Supplementary Material [file supp_3_6_418__index.html]

Aggresome formation is regulated by RanBPM through an interaction with HDAC6 — Aggresome formation is regulated by RanBPM through an interaction with HDAC6 — Supplementary Material 

# Aggresome formation is regulated by RanBPM through an interaction with HDAC6

## bio.20147021 Supplementary Material

**Files in this Data Supplement:**

- Supplementary Material - Louisa M. Salemi et al. doi: 10.1242/bio.20147021
