# Supplementary material for: Aggresome formation is regulated by RanBPM through an interaction with HDAC6
Source: Biol Open. 2014 May 2;3(6):418–30. doi: 10.1242/bio.20147021 (PMC4058076; doi:10.1242/bio.20147021)
Supplement: Supplementary Material [file supp_bio.20147021_bio.20147021-s1.pdf]

Biology Open (2014) 000, 1–13 doi:10.1242/bio.20147021

Supplementary Material
Louisa M. Salemi et al. doi: 10.1242/bio.20147021

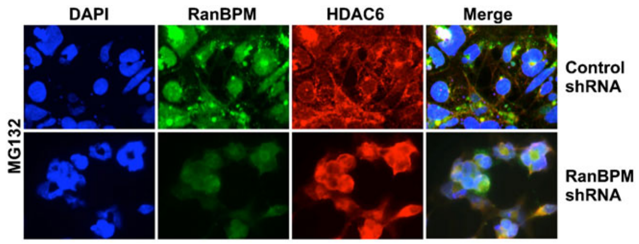

Fig. S1. Representative images of HEK293 cells stably expressing control shRNA or RanBPM shRNA treated with 5 µM MG132 fixed 16 h following MG132. Cells were processed for immunostaining with antibodies to RanBPM and HDAC6 and mounted with DAPI.

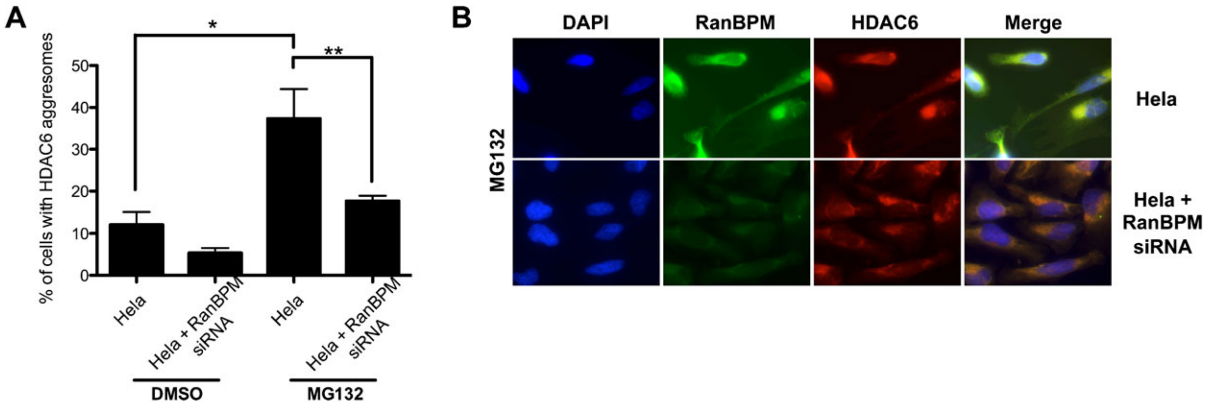

Fig. S2. Reduced aggresome formation in Hela cells transfected with RanBPM siRNA. (A) Hela cells transfected with RanBPM siRNA or left untransfected were treated with either 10 µM MG132 or DMSO and fixed 16 h following treatment. Cells were processed for immunostaining with antibodies to RanBPM and HDAC6 and mounted with DAPI. At least 50 cells were scored per experiment for the presence of RanBPM and HDAC6 aggresomes and the results are expressed as percentage of cells containing RanBPM and HDAC6 aggresomes. Results are averaged from three different experiments, with error bars indicating SE. $P<0.005$ (**); $P<0.05$ (*). (B) Representative images of Hela cells transfected with RanBPM siRNA, treated with 10 µM MG132 and processed for immunostaining as described above.

Biology Open
